# High Energy Radical Chemistry Formation of HCN-rich Atmospheres on early Earth

**DOI:** 10.1038/s41598-017-06489-1

**Published:** 2017-07-24

**Authors:** Martin Ferus, Petr Kubelík, Antonín Knížek, Adam Pastorek, John Sutherland, Svatopluk Civiš

**Affiliations:** 1 J. Heyrovský Institute of Physical Chemistry, Academy of Sciences of the Czech Republic Dolejškova 3, CZ18223 Prague 8, Czech Republic; 2 Institute of Physics, Czech Academy of Sciences, Department of Radiation and Chemical Physics, Na Slovance 1999/2, CZ18221 Prague 8, Czech Republic; 3 Medical Research Council Laboratory of Molecular Biology, Francis Crick Avenue, Cambridge Biomedical Campus, CB2 0QH Cambridge, United Kingdom

## Abstract

Recent results in prebiotic chemistry implicate hydrogen cyanide (HCN) as the source of carbon and nitrogen for the synthesis of nucleotide, amino acid and lipid building blocks. HCN can be produced during impact events by reprocessing of carbonaceous and nitrogenous materials from both the impactor and the atmosphere; it can also be produced from these materials by electrical discharge. Here we investigate the effect of high energy events on a range of starting mixtures representative of various atmosphere-impactor volatile combinations. Using continuously scanning time–resolved spectrometry, we have detected ·CN radical and excited CO as the initially most abundant products. Cyano radicals and excited carbon monoxide molecules in particular are reactive, energy-rich species, but are resilient owing to favourable Franck–Condon factors. The subsequent reactions of these first formed excited species lead to the production of ground-state prebiotic building blocks, principally HCN.

## Introduction

During the first sixteen years of the 21^st^ century, major progress in the synthesis of biological molecules under prebiotically plausible conditions has been reported^1–6^. Hydrogen cyanide (HCN) and its hydration product formamide (HCONH_2_) are central to such syntheses^5–10^. HCN exhibits many properties that make it an ideal building block, but above all it is a high energy compound – because of its triple bond – which has a propensity to form when organic matter is bombarded with excess energy^11^. Conditions on the early Earth are thought to have been harsh and so it is important to investigate the potential for HCN synthesis there. Among other high energy density events, impacts and electric discharges, which would have been common during the Hadean, can play a significant role in the synthesis of HCN^5,11–14^. Estimation of the age of craters on the Moon^15^ and the recent NICE model^16^ of the evolution of planetary orbits have both independently pointed to periods of heavy bombardments^17,18^, determined on the basis of petrographic evidence to be 4.5-3.85 ± 0.05 Gyr ago^17^. Delivery of volatiles during this bombardment^19^ would have added several reducing gases^20^ to the atmosphere produced by outgassing^21^, but impactors can also erode the atmosphere^16^ so some sort of steady state probably existed. Analysis of trace elements in a statistically significant number of ancient zircons, showing a trend of decreasing oxygen fugacity more than 3.6 Gyr ago, suggests that the early atmosphere was reducing^20^. However, the degree to which the atmosphere was reducing is still uncertain and the exact composition of the reducing mixture remains unknown. Based on astrochemical studies and analysis of interplanetary matter, we can expect delivery to the early Earth of simple molecules^19^ such as CO, NH_3_, CO_2_, H_2_O, HCN, HCHO, CH_3_OH, and maybe also HCONH_2_^22^ together with complicated substances such as polymers (tholines)^23^, various organic compounds and also biomolecules themselves^11,24–26^. All these molecules would be exposed to high energy plasma during descent and impact of their asteroidal or cometary vectors with the Earth and, if they survived this baptism of fire, to plasma created by subsequent impactors. Atmospheric components delivered by outgassing, such as CO_2_, H_2_O and N_2_, would also be exposed to these plasmas, and atoms and fragments derived therefrom would further contribute to the mix. The early atmosphere was also probably very dusty, due to volcanic activity and the ejecta of great impacts, and this dust and volcanism would have led to extensive electric discharges^14,27,28^. Furthermore, although they are not explicitly covered here, energy sources due to hydrothermalism^29^, and the strong UV radiation^11,30^ of the young Sun cannot be neglected. In previous studies, we focused on the complexifying transformations of molecules such as HCONH_2_^22^ under the harsh conditions on Earth 3.8–4.1 Gyr ago^31,32^, (i.e. only about 200 million years after cooling of the environment and formation of oceans^29^, and about 360 million years after complete surface melting of the Earth due to the moon-forming collision of Theia^15^). In the current study, we employed a wide range of experimental methods to explore HCN formation by reassembly of atoms and fragments of the components of a range of gaseous mixtures containing carbon, hydrogen, oxygen and nitrogen (C, H, O & N) exposed to conditions simulating two high energy scenarios:

- the shock wave and high energy plasma of impacting extra–terrestrial bodies into an early planetary atmosphere and
- electrical discharges of lightning in heavy clouds of dust, vapours and other aerosols from impact, volcanic activity and evaporation in the early atmosphere.

The results are discussed in relation to the molecular dynamics of reactive species rich in energy such as the ubiquitous radical ·CN and the subsequently derived HCN, which turned out to be the most important simple molecular product of recombination in plasma. The chemistry is also mapped out in connection with HCN-based synthesis of basic biomolecules. We develop the concept that some of the energy associated with the harsh conditions in which HCN is forged, inevitably then resides in this molecule, until predisposed chemistry, inevitably leading to biomolecules, dissipates it.

## Results and Discussion

To investigate the fragmentation-reassembly chemistry described above, we treated different mixtures representing various atmosphere-impactor volatile combinations in a laser spark and in a glow discharge. The reactive intermediates were monitored using optical emission spectroscopy of laser plasma and time–resolved emission spectroscopy of electric discharges. Subsequently produced simple, stable products (molecular gases) were analyzed using high–resolution rotation–vibration infrared spectroscopy.

### Decomposition of simple C, H, O & N containing atmospheres in high temperature shock waves

**Figure 1:**
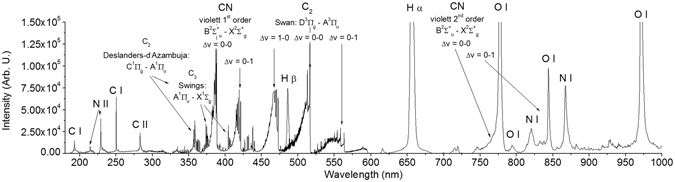
Compiled emission spectrum of laser induced breakdown in mixture of C, H, O & N bearing molecules^12,33,34,58^.

In a series of experiments, we repeatedly obtained HCN after exposing simple carbon bearing molecules such as CO, CH_4_ and CO_2_ mixed with molecular nitrogen or ammonia in presence of water, to high temperature shock waves induced by a high power laser system. The same behavior was observed starting with HCONH_2_ as a single molecular source of C, H, O & N. Figure 1 shows typical emission spectra of laser induced breakdown shock wave plasma in such simple atmospheres of C, H, O & N bearing molecules. Typical vibrational temperatures of molecular species range^12,33–35^ between 4200–6500 K while excitation temperature of atoms does not exceed 9300 K^33^. This figure is compiled from several measurements published in our previous papers so as to show typical, representative spectra^12,33,34,36^.

**Figure 2:**
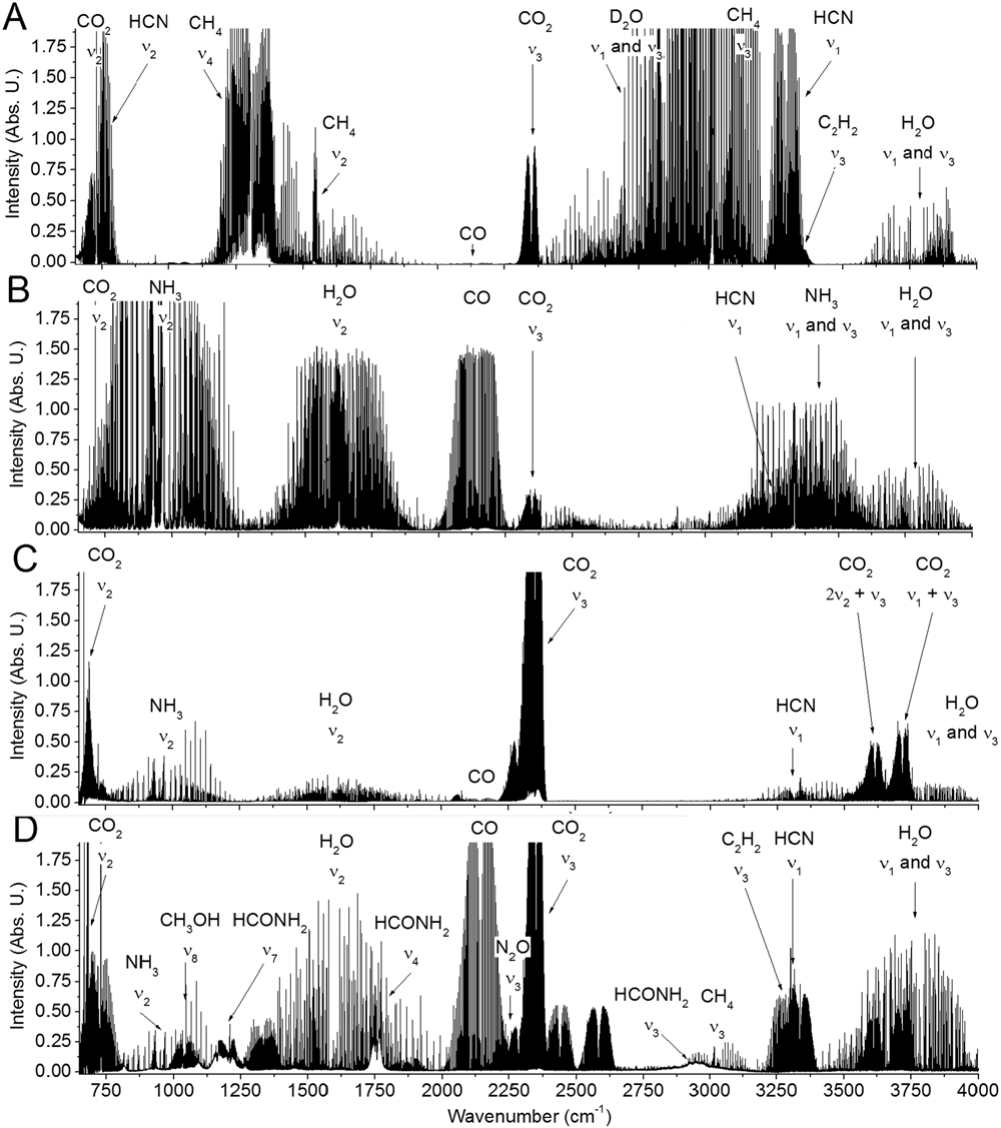
The generality of hydrogen cyanide synthesis from C, H, O & N bearing molecules exposed to high temperature shock waves. The four panels show high–resolution rotation–vibration infrared spectra of decomposition products of different mixtures exposed to laser shock waves. Panel A: CH_4_ + N_2_ + D_2_O; panel B: CO + NH_3_; panel C: CO_2_ + N_2_ + H_2_O; and panel D: HCONH_2_ + N_2_.

Among atomic ∙C, C^+^, ∙H, ∙O and ∙N, N^+^ emission lines together with blue ·C_2_ Swan emission bands, the most evident molecular bands can be assigned to the reactive ·CN radical violet rovibrational electronic transitions between excited B^1^Σ^+^_u_ and ground X^2^Σ^+^_g_ states. The ·CN radical is the presumed precursor of HCN formed in high temperature plasma^12,13,37–40^. Figure 2 shows four main examples of HCN detection in several C, H, O & N containing mixtures. The highest yields of HCN were obtained when the input carbon was in the form of CH_4_ or HCONH_2_, but significantly, even when the more stable CO & CO_2_ were the sole carbon source, HCN was still formed. Exposure of HCONH_2_ to high temperature shock waves leads to all the products recorded in previous mixtures of CO, CO_2_, CH_4_ with N_2_, NH_3_ or water. Nevertheless, the decomposition pathways of all these molecules always end with hydrogen cyanide as the most stable product in high energy plasma, as discussed in following sections.

### HCN and reactive radicals in electric discharge plasma

**Figure 3:**
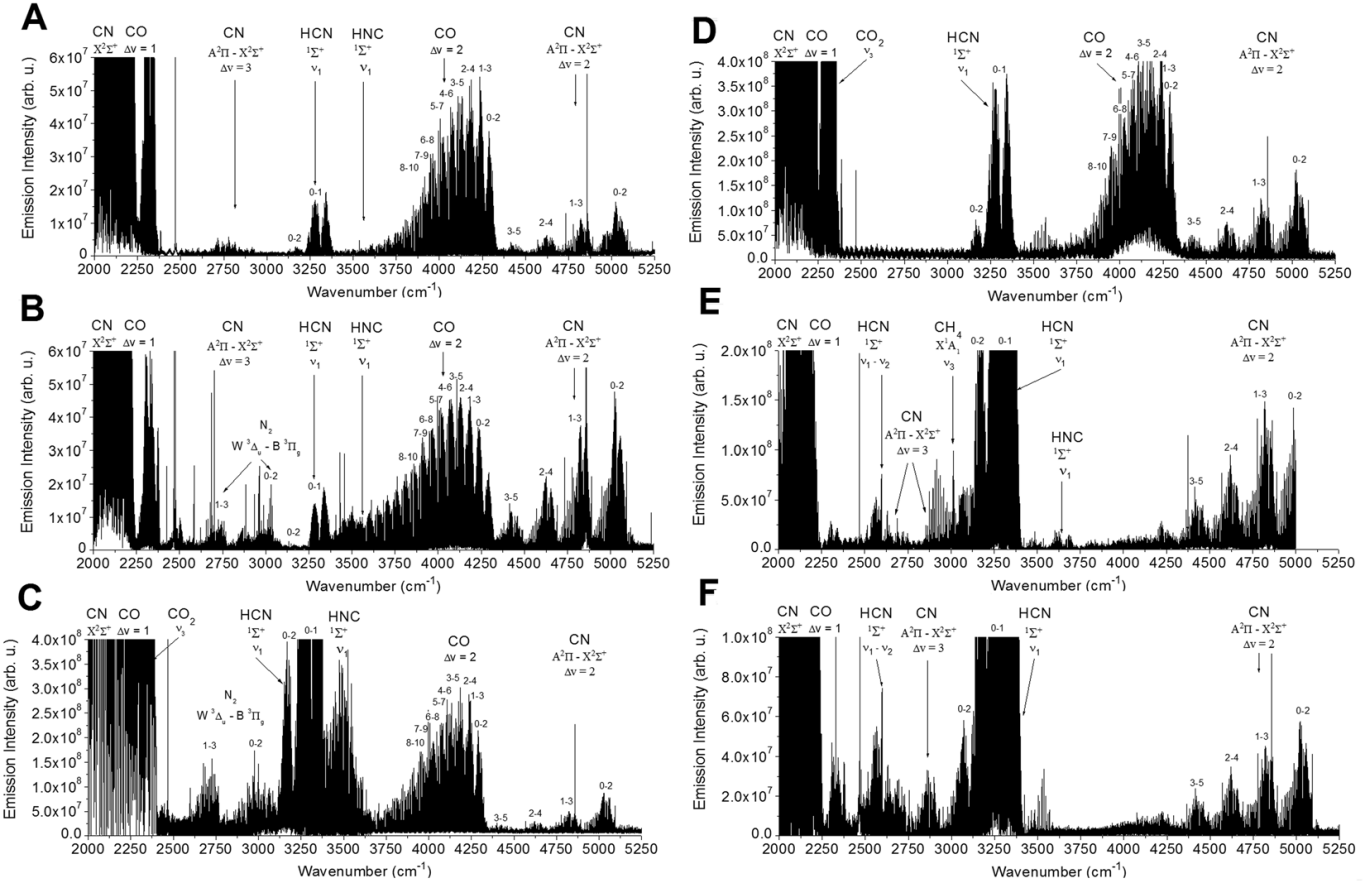
Comparison of the discharge plasma emission spectra of NH_3_: CO: H_2_O and various gas and vapour mixtures in nitrogen. Panel A shows the emission spectrum of the NH_3_ + CO + H_2_O glow discharge recorded using time–resolved spectroscopy between 1 and 5 μs after the discharge pulse. Other panels show emission spectra of different mixtures in a similar time domain: Panel B: HCONH_2_ + N_2_ + H_2_O; Panel C: HCHO + N_2_ + H_2_O; Panel D: CH_3_OH + N_2_ + H_2_O; Panel E: CH_4_ + N_2_ + H_2_O; and Panel F: CH_3_CN + N_2_ + H_2_O.

In the same way that laser induced breakdown was monitored, spectra of unstable radicals in discharge plasma were observed using emission spectroscopy. However, our time–resolved Fourier transform high resolution interferometer (ref.^41^ and references therein) allows monitoring in the infrared range on the microsecond time scale. In our previous work^13,41^, we have already showed the kinetic behaviour of several radicals and unstable species including ·CN, ·C_2_, ·CH and others. In the current study, we demonstrate that various mixtures containing sources of C, H, O & N always produce the ·CN radical together with a high yield of HCN. In Fig. 3, the discharge emission spectra of various C, H, O & N containing mixtures are compared. The samples include prebiotically relevant molecules such as formamide, formaldehyde, methanol, methane and acetonitrile that are found in comets or produced by atmospheric photochemistry. Looking at the discharge spectra of samples in generally reducing atmospheres, several species that are very similar to the discharge chemistry of pure formamide (containing C, H, O, & N in the same molecule) can be observed. However, this spectrum is rich in other dissociation fragments, such as ∙NH and ∙CH. In all the spectra, the most prominent bands belong to CO, CO_2_, HCN and the unstable radical ∙CN^5,7,42,43^. Specifically, the main species and their detected states in the emission spectra are the following: A^2^Π − X^2^Σ^+^ Δv = 2, 3 and rotation vibration bands of the ground electronic state X^2^Σ^+^ Δv = 1 ∙CN; X^3^Σ^−^ rotation−vibration lines of ∙NH; ^2^Π of the radical ∙CH (very weak but detectable only in formamide) and stable products such as the *v*_*1*_ band of HCN and its unstable isomer^13^ HNC and a series of the rows of very strong highly excited rotational−vibrational transitions of CO together with strong atomic emission lines C I (e.g., strong line ^3^P^0^−^3^D, 2684.51 cm^−1^); H I (very strong infrared α−Brackett series approximately 2469 cm^−1^); O I (e.g., weaker line ^5^D^0^−^5^F 2576.14 cm^−1^); N I (e.g., weaker line ^2^D^0^−^2^F 3109.35 cm^−1^); and, finally, the infrared electronic transition W^3^Δ_u_ –B^3^Π_g_ of N_2_.

### Radical chemistry

**Figure 4:**
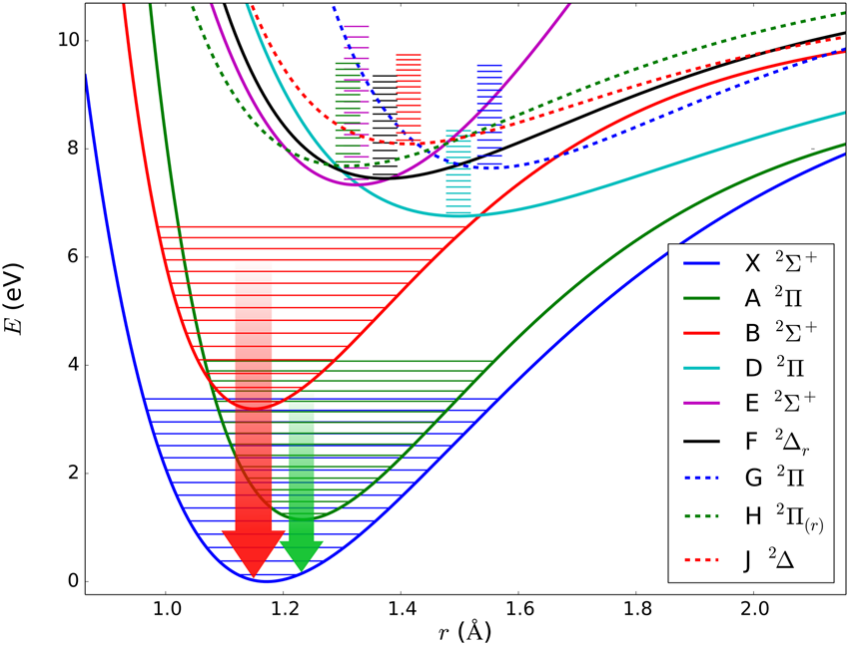
Potential energy surfaces for ∙CN radical in several electronic excited states. The electronic ro-vibrational transitions observed using infrared emission time resolved spectroscopy of electric discharge and optical emission spectroscopy of laser plasma are marked in green and red respectively.

**Figure 5:**
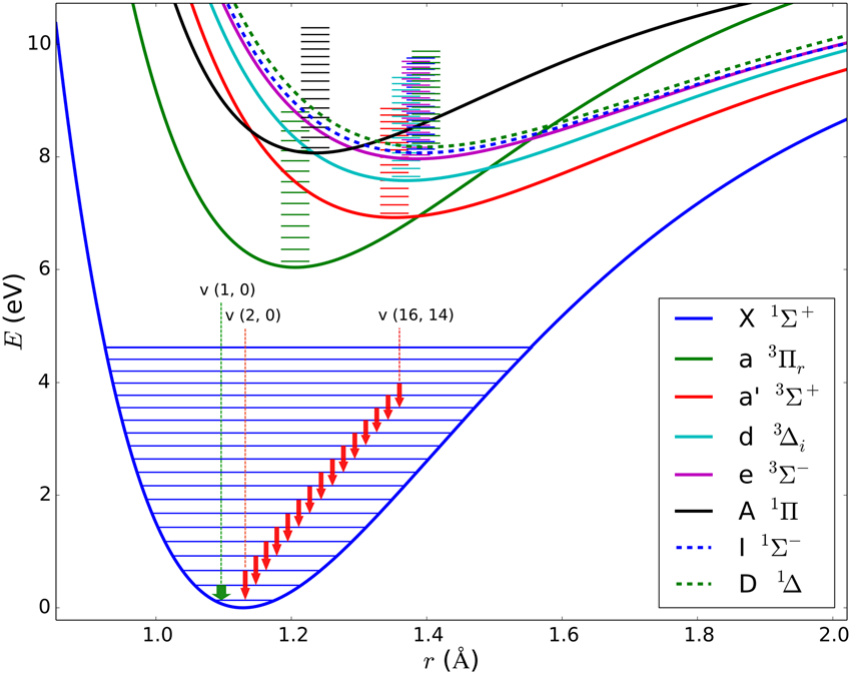
Potential energy surfaces for CO in several excited electronic states. The rotational-vibration transitions together with the second harmonic band transition have been observed up to vibrational level v = 16 (inside of fundamental X^1^Σ^+^ electronic state) using infrared emission time resolved spectroscopy of electric discharge are marked in green and red respectively.

The radical ∙CN together with the most stable molecules such as CO and HCN were detected in all the studied C, H, O & N containing mixtures. With respect to the detection of formamide particularly among CO + NH_3_, H_2_O discharge products, we also expect ∙CH and ∙NH in all the mixtures, but these were probably below the detection limit. These radicals play a very important role in plasma chemical reactions^5,10,44^. However, the ∙CN radical and the vibrationally excited CO molecule are dominant in the emission spectra of the studied reduction mixtures. The ∙CN radical, due to its very rigid structure (strong triple bond) and rather complicated electronic and rovibrational spectrum, allows transitions between a whole series of excited states, such as A^2^Π − X^2^Σ^+^ Δv = 2, 3; the ground state X^2^Σ;^+^ violet system B^1^Σ^+^_u_ − X^2^Σ^+^_g_ in laser induced plasma; etc. The first 9 states are shown schematically in the Fig. 4. Potential surfaces have been schematically plotted using constants taken from ref.^45^. Due to very favorable Frank−Condon factors, the ∙CN radical can be excited to very high vibrational and rotational states, which results in observations of a vast range of transitions from the visible spectrum through the infrared to the microwave regions^3,5,10,13,33,36,43,44,46–50^. Together with this capability, this reactive species exhibits high stability due to bond dissociation energy of 7.77 eV (62 711 cm^−1^)^51^. The ∙CN radical is thus capable of absorbing a huge amount of energy and populating a great number of energy levels without dissociating. Due to this effect, this species can interact (transfer energy) with practically any molecule or radical in a broad spectral range from the ultraviolet up to the microwave region. The resilience of this radical and its common acquisition of a hydrogen atom to generate HCN mean that the latter species is an inherently formed product of high energy chemistry in C, H, O & N containing atmospheres. Another highly energetic particle is the CO molecule. The spectra in all figures except those in Fig. 3 panel E and F, which correspond to methane and acetonitrile discharges, show the overtones of the CO molecule excited to high vibrational states (up to v* = *16 here). The corresponding transitions are depicted schematically in the Fig. 5. In the high energy plasma, the ∙CN radical and CO molecule are unique in that they are able to store thermal energy otherwise dissipated in a multi−step reaction via excitation of their numerous vibrational and rotational transitions. They thereby control the energy flow through the whole reaction pathway.

Among the species identified in the discharge products of all the mixtures using absorption spectroscopy were ammonia, carbon monoxide, acetylene, nitric oxide and hydrogen cyanide. Acetonitrile and methane discharges in the presence of nitrogen exhibited the formation of acetonitrile from methane and methane from acetonitrile. In the mixture representing the pure reducing atmosphere containing CO, NH_3_, and H_2_O, we also detected large amounts of formamide, as described in the following sections. In all the experiments, HCN together with the ∙CN radical is ubiquitous. We can assume, that the stable initial products of discharge plasma in all studied mixtures such as HCN, CO, and NH_3_, subsequently react with each other to a small extent, together with ∙CN and other radicals (∙OH, ∙NH), thus producing larger stable molecules.

It can be assumed that the ∙CN radical is formed in several different channels in high energy plasma. In our previous work^12^, we demonstrated, using isotopically labeled water, that HCN is produced in methane-nitrogen mixtures exposed to shock waves mainly by the following reaction:

1 $$2{{\rm{CH}}}_{4}+{{\rm{N}}}_{2}\to 2{\rm{HCN}}+3{{\rm{H}}}_{2}$$

We discovered that participation of hydrogen released from water is negligible. This fact was demonstrated by the absence of DCN in the mixture of CH_4_ + D_2_O + N_2_. In fact, this chemistry can be explained similarly with the assumed mechanism of HCN synthesis in the atmosphere of Titan^52^. The proposed mechanism can be broken down into fundamental steps as follows: Dissociation of N_2_ leads to the formation of an N∙ radical, an electron and an N^+^ ion:

2 $${{\rm{N}}}_{2}\to {{\rm{N}}}^{+}+{{\rm{e}}}^{-}+{\rm{N}}\cdot $$

or regarding common radical mechanism, to formation of two nitrogen radicals. This option represents energetically more favorable channel^13^, because energy is not consumed both for dissociation and ionization:

3 $${{\rm{N}}}_{2}\to {\rm{N}}\cdot +{\rm{N}}\cdot $$

However, we should note that in the emission spectra of laser shock wave plasma depicted in the Fig. 1, N∙ radical (in spectroscopic notation N I) as well as N^+^ ion (in spectroscopic notation N II) can be found. It can be assumed that subsequently, the N^+^ ion reacts with methane producing an H_2_CN^+^ ion and two hydrogen atoms:

4 $${{\rm{N}}}^{+}+{{\rm{CH}}}_{4}\to {{\rm{H}}}_{2}{{\rm{CN}}}^{+}+2{\rm{H}}\cdot $$

The H_2_CN^+^ ion then combines with an electron and is thereby decomposed to HCN and a hydrogen atom:

5 $${{\rm{H}}}_{2}{{\rm{CN}}}^{+}+{{\rm{e}}}^{-}\to {\rm{HCN}}+{\rm{H}}\cdot $$

However, we should note that H_2_CN^+^ ion is not detected in our emission spectra. N radical is very reactive species^53^. It can be assumed that this particle reacts with a ∙CH_3_ radical produced by C–H homolysis of methane:

6 $$\cdot {{\rm{CH}}}_{3}+{\rm{N}}\cdot \to {\rm{HCN}}+{{\rm{H}}}_{2}$$

or directly with methane molecule:

7 $${{\rm{CH}}}_{4}+{\rm{N}}\cdot \to {\rm{HCN}}+{{\rm{H}}}_{2}+{\rm{H}}\cdot $$

These various steps sum to the stoichiometry given by Equation (1). In fact, our results show that in the case of a methane and nitrogen containing atmosphere, the ∙CN radical together with HCN can be assumed as terminal products of this fundamental ion-radical chemistry whether energy is supplied photochemically as on Titan, or, on early Earth (where the methane could have been delivered by cometary impact)^19^, by lighting or shock waves induced by impactors.

On the other hand, in carbon monoxide-ammonia mixtures exposed to shock waves, HCN formation can be partly explained by initial synthesis of formamide:

8 $${{\rm{NH}}}_{3}+{\rm{CO}}\to {{\rm{HCONH}}}_{2}$$

Formamide then undergoes dehydration to HCN^54^:

9 $${{\rm{HCONH}}}_{2}\to {{\rm{H}}}_{2}{\rm{O}}+{\rm{HCN}}$$

A second route to HCN in carbon monoxide-ammonia mixtures exposed to shock waves involves reaction of CO with molecular nitrogen^55^. In their work, formation of diazirinone N_2_CO, isocyanato radical OCN and NCN radical was observed alongside with nitric oxide NO and nitrogen dioxide NO_2_. In our emission and absorption spectra of shock waves or electric discharges, we have observed formation N_2_O together with CN and HCN. N_2_CO, OCN or HOCN, HNCO isomers as well as NCN species have not been detected, NO is observed in traces near the detection limit (less than 10 ppmV). It can be assumed that shock wave chemistry preferably starts with reaction of CO with N radical:

10 $${\rm{CO}}+{\rm{N}}\to {\rm{OCN}}$$

which is in equilibrium process quickly decomposed to either CO or CN:

11 $${\rm{OCN}}\to {\rm{CO}}\,{\rm{or}}\,{\rm{CN}}$$

CN reacts with H generated from water and producing HCN:

12 $${\rm{CN}}+{\rm{H}}\to {\rm{HCN}}$$

While NO, NO_2_ and N_2_O can be produced by reaction with O radical:

13 $${\rm{N}}+{\rm{O}}\to {\rm{NO}}$$

14 $${\rm{O}}+{{\rm{N}}}_{2}\to {\rm{NO}}+{\rm{N}}$$

15 $${\rm{NO}}+{\rm{O}}\to {{\rm{NO}}}_{2}$$

16 $${\rm{NO}}+{\rm{NO}}\to {{\rm{N}}}_{2}{\rm{O}}+{\rm{O}}$$

In our experiments, we usually observe N_2_O as the most stable product under plasma conditions. NO together with NO_2_ are usually observed in traces. The reactions (13), (14) and (15) have been proposed in study of Kaiser *et al*.^55^ and reaction (16) is presented in work of Zeldovitch^56^. We assume that the reaction (16) explains why the absorption spectra reported in this study exhibit strong absorption bands of N_2_O. The rich chemistry described above certainly merits further investigation by theoretical quantum chemistry calculations, kinetic modeling and in depth experimental exploration of all the mentioned systems.

## Conclusions

Excited species CN radical and excited CO play dominant role either in high temperature shock wave or in discharge chemistry in mixtures containing C, H, O, and N compounds. The ∙CN radical and CO molecule are unique in that they are able to store a huge amount of thermal energy that less stable entities would otherwise dissipate by dissociation and multi–step reactions. The resilience of the ∙CN radical and CO molecule, due to strong triple bonds and excitation of numerous vibrational and rotational transitions, thereby controls the energy flow in high energy C, H, O & N containing chemical systems. The stable products in all the studied mixtures are HCN, CO, and NH_3_. Subsequent reactions of these species with each other and with ∙CN and other radicals (∙H and ∙NH_x_) do occur to some extent and produce a few larger molecules some of which are relevant in the context of the origin of life, but yields are low^10^. However, in this context, the efficient production of HCN in high energy chemistry of C, H, O & N containing mixtures of a wide range of composition, and its survival after cessation of high energy input are the most striking findings. Together they suggest that HCN would have been continuously produced and maintained during impact and discharge events on the early Earth. However, in the absence of a mechanism for accumulation of HCN or derivatives thereof on the ground, atmospheric erosion would have kept HCN levels at a steady state^16^. HCN can be extremely efficiently sequestered in aqueous solution by reaction with ferrous ions giving ferrocyanide^6^. The extremely favourable equilibrium constant for the formation of ferrocyanide means that Henry’s law limitations that would normally apply to the absorption of atmospheric HCN into water are overcome. Evaporation of solutions of ferrocyanide salts generated by weathering in this way would have accumulated and concentrated HCN as these coordination compounds, but a means of recovering their organic component for subsequent prebiotic synthesis would have been necessary. Simple heating of the ferrocyanide evaporite residue – either through impact or geothermalism – would have sufficed in this regard as it is known that thermal decomposition of ferrocyanide salts produces non-coordination products the nature of which depends on the cation(s) associated with the ferrocyanide^6^. Thus, magnesium nitride, calcium cyanamide, calcium carbide & sodium and potassium cyanide can be generated. Dissolution of these materials in water generates the starting materials needed to furnish all carbon and nitrogen atoms of nucleotides, amino acids and lipids through reductive homologation chemistry^6^. The synthesis that ensues is written into the chemistry of cyanide just as its formation and persistence are. The synthesis produces a palette of compounds that corresponds to building blocks for further chemistry and the emergent phenomenon of life.

The foregoing describes the chemistry from a material perspective, but it also pertinent to consider it from an energy perspective as the reductive homologation chemistry relies on two sources of energy: UV photons drive the production of hydrated electrons from certain anions; and the triple bonds of hydrogen cyanide and other nitriles that derive from it provide the chemical energy to drive addition reactions. Delivery of C, H, O & N containing volatiles to the surface of the early Earth and electrical discharge led to ferocious conditions that rent them and outgassed atmospheric components assunder. During subsequent high energy chemistry, cyanide radicals formed and persisted due to an inherent resilience to such conditions. The strong triple bond of the cyanide radical, in part responsible for this resilience, effectively stores some of the energy of the radical’s violent creation. As conditions abate and time passes, cyanide radicals become hydrogen cyanide and then ferrocyanide salts, but the triple bond and its stored energy endure all the while. Then high energy chemistry again takes place as the ferrocyanide salts are thermally decomposed – but still triple bonds persist. Finally, the thermal decomposition products become dissolved in water, and are subject to UV irradiation and then a large portion of the triple bonds succumb to chemistry that is driven by their stored energy. Life can thus be viewed as a consequence of the chemical reactivity, and energy storage capacity, of hydrogen cyanide under planetary conditions such as those undergone by Earth 3.8–4.1 Gyr ago.

## Materials and Methods

### Laser shock wave plasma experiments

To explore the chemistry of simple C, H, O & N mixtures in shock–wave plasma, we treated different reaction mixtures representing a reducing atmosphere with a large laser spark. The laser beam is be focused in the center of the 25-L sample cell by a plano-convex lens, with a diameter of 20 cm and a focal length of 25 cm. During the dielectric breakdown in gas (LIDB) generated with a laser pulse of energy 150 J (time interval ≈350 ps, wavelength ≈1.315 μm) all processes connected with a high−energy density take place: shock rise in temperature to several thousand K, the formation of a shock wave and the generation of secondary hard radiation. Using our optical set–up, a total of 50 laser pulses were applied with a concentrated energy of 7500 J with peak power of 4.3 × 10^10^ W. The reactive intermediates are monitored *in situ* using optical emission spectroscopy; simple molecular gases were detected by means of infrared absorption spectroscopy. Laser Shock Wave Plasma experiments have been carried out using the high–energy laser PALS (Prague Asterix Laser System) using small one liter cells filled with the reactants: mixtures of ammonia, carbon monoxide, carbon dioxide and nitrogen in the gas phase, which were purchased from Linde Gas, and water, CHROMASOLV® HPLC grade, which was purchased from Sigma Aldrich, at 760 Torr of gases in the presence of 10 mL of liquid water.

### Optical emission spectroscopy of laser induced breakdown plasma

For the static LIDB experiments, a 25-L glass cell was used. The shape of the cell body was a cross with both length and width of 40 cm, with four windows. The main laser-beam entrance windows were made of 4 cm thick glass, with a diameter of 20 cm. Additional 1.5 cm thick inspection windows with a diameter of 10 cm served for optical emission signal (OES) detection. The windows were mounted to the glass body with stainless-steel flanges and sealed with Viton rings. The cell was mounted on a 1 cm thick aluminum plate for easier manipulation and positioning. The cell’s body was equipped with two vacuum valves (ACE Glass Inc., Vineland USA) for gas handling. The emission spectra were measured *in situ* during the irradiation experiment. The optical emission signal (OES) was focused directly (without the lens) on the entrance slit of the spectrometer. Two ruled gratings were switched in the spectrometer (MS257, Oriel), one for preview (150 lines/mm, width of the measured spectra = 400 nm) and the second for a high-resolution measurement (1200 lines/mm, width of the measured spectra = 60 nm). The dispersed spectrum was detected using an intensified CCD camera (ICCD; iStar 720, Andor) with a resolution of 0.08 nm/pixel for the high-resolution grating. Spectral lines at a distance down to 0.2 nm have been distinguished using a very narrow entrance slit to prevent saturation of the camera. The synchronization of intensified CCD camera with the laser pulse was accomplished using a signal from the photodiode. Each of the presented spectra corresponds to the gas as it is exposed to a single laser shot. Because of observed electrical interference on the ICCD head occurring in the high-power laser hall, the spectrometer was placed in the Faraday cage. The synchronization of the laser and the ICCD detector allowed us to obtain information on the emission signal in a time resolved fashion. Due to the use the of high-resolution dispersion grating, we were able to obtain a 60 nm wide spectrum from a single measurement. For that reason, the general view spectra are composed from several narrower (60 nm) interconnected spectral intervals. These intervals corresponded to the individual laser pulses, whose energy, after subtracting for losses, was approximately 150 J.

### Discharge experiments

For the study of the chemistry of small radicals^5,7,42,43^, we have developed a time–resolved technique on the microsecond time scale, together with high–resolution Fourier transform spectroscopy controlled by a Field–Programmable Gate Array processor (FPGA) microcontroller. The main role of the FPGA processor in our experiment was to create a discharge or laser pulse and AD trigger signals (the signal for data collection from the detector) synchronously with the HeNe laser fringe signals from the interferometer. The FPGA processor also controls the data transmission from the digital input board to the PC. The use of time–resolved FT spectroscopy has opened new pathways and new points of view in the study of the formation and decay processes inside the discharge plasma and permits description of the dynamics of the formation and decay of excited states of atoms, radicals and ions inside a reducing atmosphere of plasma. Here, we are able to study individual processes using atomic or molecular lines in a wide spectral range of high–resolution FT technology, which is also extended into the time dimension. Discharge experiments have been conducted in a glow–discharge emission cell for spectroscopic monitoring of unstable products using time–resolved spectroscopy. The principles of the method have been discussed in our previous papers^13,41,57^. Briefly, a mixture of the gases and the precursor molecules (CH_4_, CO, HCONH_2_, HCHO, CH_3_OH or CH_3_CN) in nitrogen buffer gas or ammonia was directed to a positive column discharge tube equipped with CaF_2_ windows. The emission spectra from the discharge plasma in different gas mixtures were observed with the time–resolved Fourier transform high–resolution Bruker IFS 120 HR interferometer. The AC discharge was maintained by a high–voltage transistor switch HTS 81 (Behlke electronic GmbH, Frankfurt, Germany) and applied between the stainless steel anode and the grounded cathode. The plasma was cooled by liquid water in the outer jacket of the cell. The voltage drop across the discharge was 1000 V, with a pulse width of 22 μs and 0.6 A peak–to–peak current. The scanner velocity of FTS was set to produce a 10 kHz HeNe laser fringe frequency, which was used to trigger the discharge pulse. The recorded spectral range was 1800–4000 cm^−1^ with an optical filter, at an unapodized resolution of 0.02 cm^−1^. A total of 50 scans were averaged to obtain a reasonable signal–to–noise ratio. The initial pressure was changed from 1 to 10.0 Torr.
