# Author Correction: High Energy Radical Chemistry Formation of HCN-rich Atmospheres on early Earth

**DOI:** 10.1038/s41598-018-21909-6

**Published:** 2018-03-06

**Authors:** Martin Ferus, Petr Kubelík, Antonín Knížek, Adam Pastorek, John Sutherland, Svatopluk Civiš

**Affiliations:** 1 J. Heyrovský Institute of Physical Chemistry, Czech Academy of Sciences, Dolejškova 3, CZ18223 Prague 8, Czech Republic; 2 Institute of Physics, Czech Academy of Sciences, Department of Radiation and Chemical Physics, Na Slovance 1999/2, CZ18221 Prague 8, Czech Republic; 3 Medical Research Council Laboratory of Molecular Biology, Francis Crick Avenue, Cambridge Biomedical Campus, CB2 0QH Cambridge, United Kingdom

Correction to: *Scientific Reports* 10.1038/s41598-017-06489-1, published online 24 July 2017

In the original version of this Article, an affiliation for Petr Kubelík was omitted. The correct affiliations for Petr Kubelík are listed below:

J. Heyrovský Institute of Physical Chemistry, Czech Academy of Sciences, Dolejškova 3, CZ18223, Prague 8, Czech Republic.

Institute of Physics, Czech Academy of Sciences, Department of Radiation and Chemical Physics, Na Slovance 1999/2, CZ18221 Prague 8, Czech Republic.

